# The ChickenGTEx portal: a pan-tissue catalogue of regulatory variants shaping transcriptomic and phenotypic diversity

**DOI:** 10.1093/nar/gkaf731

**Published:** 2025-08-19

**Authors:** Yali Hou, Dong Zou, Qin Chu, Bujie Zhan, Ruizhen Wang, Dailu Guan, Wannian Wang, Xiao Feng, Xin Li, Xiaoning Zhu, Zhonghao Bai, Yahui Gao, Hongwei Yin, Tianyi Xu, Zhixiang Yuan, Xiaoxiang Hu, Ning Yang, Huaijun Zhou, Lingzhao Fang, Zhang Zhang

**Affiliations:** State Key Laboratory of Animal Biotech Breeding; National Germplasm Center of Domestic Animal Resources, Institute of Animal Science, Chinese Academy of Agricultural Sciences, Beijing 100193, China; National Genomics Data Center, China National Center for Bioinformation, Beijing 100101, China; Beijing Institute of Genomics, Chinese Academy of Sciences, Beijing 100101, China; Institute of Animal Husbandry and Veterinary Medicine, Beijing Academy of Agriculture and Forestry Sciences, Beijing 100097, China; Department of Basic Medical Sciences, Medical College, Longdong University, Qingyang 745000, China; Academy of Chinese Medical Sciences, Henan University of Chinese Medicine, Zhengzhou 450046, China; Department of Animal Science, University of California, Davis, CA 95616, United States; Department of Animal Science, Shanxi Agricultural University, Taigu 030801, China; College of Animal Science and Technology, China Agricultural University, Beijing 100193, China; State Key Laboratory of Animal Biotech Breeding; National Germplasm Center of Domestic Animal Resources, Institute of Animal Science, Chinese Academy of Agricultural Sciences, Beijing 100193, China; College of Animal Science and Technology, China Agricultural University, Beijing 100193, China; State Key Laboratory of Animal Biotech Breeding, College of Biological Sciences, China Agricultural University, Beijing 100193, China; State Key Laboratory of Livestock and Poultry Breeding, Guangdong Provincial Key Lab of Agro-Animal Genomics and Molecular Breeding, College of Animal Science, South China Agricultural University, Guangzhou 510642, China; Shenzhen Branch, Guangdong Laboratory of Lingnan Modern Agriculture, Key Laboratory of Livestock and Poultry Multi-omics of MARA, Agricultural Genomics Institute at Shenzhen, Chinese Academy of Agricultural Sciences, Shenzhen 518124, China; National Genomics Data Center, China National Center for Bioinformation, Beijing 100101, China; Beijing Institute of Genomics, Chinese Academy of Sciences, Beijing 100101, China; National Genomics Data Center, China National Center for Bioinformation, Beijing 100101, China; Beijing Institute of Genomics, Chinese Academy of Sciences, Beijing 100101, China; State Key Laboratory of Animal Biotech Breeding, College of Biological Sciences, China Agricultural University, Beijing 100193, China; College of Animal Science and Technology, China Agricultural University, Beijing 100193, China; Department of Animal Science, University of California, Davis, CA 95616, United States; Center for Quantitative Genetics and Genomics (QGG), Aarhus University, Aarhus 8000, Denmark; National Genomics Data Center, China National Center for Bioinformation, Beijing 100101, China; Beijing Institute of Genomics, Chinese Academy of Sciences, Beijing 100101, China

## Abstract

A systematic dissection of the functional impacts of non-coding variations across diverse tissues and cell types is essential for deciphering the molecular architecture underlying complex traits. Given the significance of chickens as both a key livestock species and a fundamental model organism, the development of an integrative genomics resource is imperative. Leveraging SNP-to-gene-to-trait linking strategies—including molecular quantitative trait loci (molQTL), regulatory elements, and context- or environment-dependent regulatory heterogeneity—we developed the ChickenGTEx portal (http://chicken.farmgtex.org), which provides a comprehensive catalogue of regulatory effects on transcriptomic and phenotypic diversity across tissues, cell types, and sexes. Key features of the resource include a genotype imputation panel of 2869 chickens from 123 breeds worldwide, five types of molecular phenotypes across 28 tissues, ∼2.2 million molQTL, 806 229 fine-mapped molQTL, 1956 context-dependent molQTL, 257 genome-wide profiles of 7 epigenetic marks (representing 15 chromatin states) from 23 tissues, 185 376 single-cell expression profiles across 191 cell clusters from 9 tissues, and 96 386 gene-trait associations covering 108 economically important traits. In summary, the ChickenGTEx portal will serve as an invaluable resource for advancing research in fundamental and evolutionary biology, chicken precision breeding, and eventually human biomedicine.

## Graphical Abstract

**Graphical Abstract:**
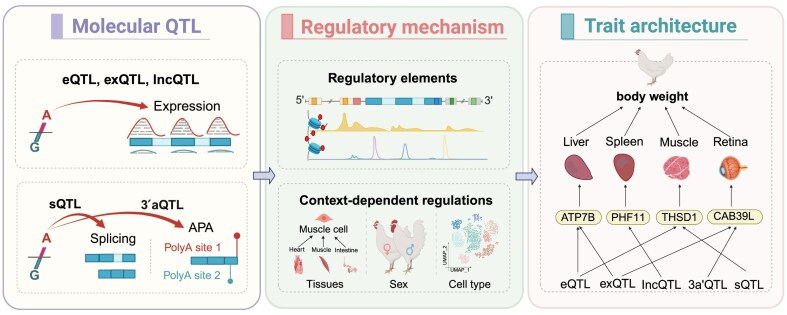

## Introduction

Over the past decade, advancements in high-throughput sequencing technologies and genome-wide association studies (GWAS) have revolutionized our understanding of the genetic architecture (e.g. causal variants, their frequency, and effects) underpinning complex traits and adaptive evolution across diverse organisms [1–5]. Notably, over 80% of trait-associated variants reside in non-coding regions, where they play pivotal roles in regulating genomic biology (e.g. chromatin organization and gene expression), ultimately influencing phenotypic variation and disease susceptibility [6]. Accurate annotation of non-coding variants is crucial for deciphering the molecular mechanisms underlying complex traits. To meet this need, large-scale consortia such as the Encyclopedia of DNA Elements (ENCODE) [7] and the Genotype-Tissue Expression (GTEx) project [8] have systematically integrated multi-omics data to map regulatory elements and assess the functional impact of non-coding variation primarily in humans and mice. These efforts have yielded a suite of influential resources and web-based tools—including RegulomeDB [9], HaploReg [10], SCREEN [11], FunSeq [12], and GTExPortal [8]—that facilitate the interpretation of non-coding variants identified through genome sequencing and GWAS, thereby advancing our understanding of the regulatory mechanisms underlying complex traits and diseases.

Farm animals, alongside humans and classical model organisms, serve as indispensable resources for biological research due to their well-controlled sampling conditions and experimental accessibility [13]. Investigating the functions of non-coding variants in these species carries profound implications for sustainable agriculture, basic biological insights, and human biomedicine advancements [14–16]. The Farm Animal Genotype-Tissue Expression (FarmGTEx) Project [13], in conjunction with the Functional Annotation of Animal Genomes (FAANG) initiative [17], was launched to investigate how non-coding variants regulate gene expression across tissues and complex traits in domestic animals. Chickens are a vital source of high-quality protein in human diets and exhibit unique characteristics in development, evolution, genomics, physiology, and immunity, accordingly serving as a fundamental model organism [18, 19]. Despite being the first sequenced farm animal and avian species, systematic characterization of chicken regulatory variants and their effects on transcriptomic and phenotypic variations is still lacking.

To bridge this gap, the Chicken Genotype-Tissue Expression (ChickenGTEx) project, part of the FarmGTEx initiative, was launched to identify and characterize molecular quantitative trait loci (molQTL) that regulate transcriptomic and phenotypic diversity across various biological contexts, including tissues, cell types, genetic backgrounds, and sexes [14]. In parallel, a comprehensive pan-tissue atlas of regulatory elements has been established in chickens, providing foundational insights into *cis-*regulatory landscapes [20]. Recently, several studies have begun to uncover single-cell expression heterogeneity in chicken tissues [21–27]. Despite these advances, there remains a pressing need for an integrative framework that unifies regulatory variants, functional annotations, and trait associations to decipher the genetic architecture of complex traits in chickens. Herein, we developed the ChickenGTEx portal (http://chicken.farmgtex.org), a comprehensive and user-oriented platform built upon a robust variant-to-gene-to-trait (V2G2T) linking framework that incorporates molQTL, regulatory elements, and context-dependent regulatory heterogeneity. This resource systematically catalogues the regulatory impacts of genomic variants on transcriptomic and phenotypic variation, thereby providing a powerful foundation for dissecting gene regulation, revealing fundamental biological principles, and accelerating precision breeding and translational advances in agriculture and beyond.

## Materials and methods

### Data information and curation

Leveraging a V2G2T framework, the ChickenGTEx portal integrates pan-tissue molQTL [14], regulatory elements [20], single-cell expression landscapes [21–27], and context-dependent regulatory variations [14]. This integrative platform empowers users to access, visualize, and utilize the resource with ease, while systematically deciphering the molecular and cellular blueprints underlying complex traits and phenotypic diversity in chickens.

The portal hosts detailed metadata for 7015 RNA sequencing (RNA-seq) and 2869 whole-genome sequencing (WGS) datasets, including information on BioProject, BioSample, breed, age, sex, sequencing platform, read depth, data quality, and alignment statistics. The RNA-seq datasets encompass 52 distinct tissues grouped into 28 tissue categories—most notably liver (12%), muscle (8%), and spleen (7%)—collected from 113 chicken breeds, including Leghorn (24%), Ross Rhode (7%), and Island Red (5%). Low-quality reads were trimmed using Trim Galore (v.0.6.6), and the clean reads were aligned to the GRCg6a reference genome using the STAR (v.2.7.7a) algorithm, resulting in an average uniquely mapping rate of 91% that ranges from 60% to 99% [14]. The WGS datasets span 153 genetically and geographically diverse breeds [14], such as Rhode Island Red (16%), Yellow-plumage Dwarf (6.8%), Leghorn (5%), Tibetan (4%), and COBB (3%), with an average sequencing depth of 12.5× and an average mapping rate of 98%. In addition, the portal incorporates 257 epigenomes across 23 tissues—including cortex, hypothalamus, cerebellum, testis, muscle, heart, adipose, kidney, spleen, bone marrow, lung, trachea, thymus, liver, proventriculus, gizzard, duodenum, jejunum, ileum, colon, cecum, shell glands, and bursa—each derived from 2 to 4 chickens [20]. Furthermore, the portal integrates single-cell transcriptomic profiles from nine diverse tissues—liver, spleen, heart, breast muscle, bursa of Fabricius, retina, lung, amygdala, and mesencephalon [21–27].

### Characteristics of five types of molecular phenotypes

The methods involved in the portal were concisely summarized here for convenience. Five transcriptomic phenotypes were identified from the RNA-seq datasets: protein-coding gene (PCG) expression, exon expression, long non-coding RNA (lncRNA) expression, 3′UTR alternative polyadenylation (APA), and alternative splicing variation (ASV) across 28 different tissues. Leveraging the alignment files, the gene expression (i.e. transcripts per million, TPM) of PCGs and lncRNAs was calculated with featureCounts (v.2.0.1) [28] and StringTie (v.2.1.5) [29]. Their tissue expression pattern (i.e. tissue-specificity) was evaluated using eight scoring metrics (i.e. Counts, Tau, Gini coefficient, Simpson index, Shannon entropy specificity, ROKU specificity, Specificity measure dispersion, and Jensen-Shannon specificity dispersion), implemented in the tspex package, where 0 indicates broad expression and 1 indicates tissue-specific expression [30]. Exon expression was estimated using featureCounts (v.2.0.1) [28] and TBtools (v.1.09) [31]. APA events were identified using the DaPars (v.2) algorithm [32, 33], where the percentage of distal poly(A) site usage index (PDUI) value for each gene in each sample was calculated. ASVs, focusing on intron excisions, were identified using the LeafCutter package [34], where the percent spliced-in (PSI) value for each cluster of introns in each sample was calculated.

### Identification of molQTL associated with five types of molecular phenotypes

A high-quality genotype imputation panel was established using the WGS data of 2869 chickens worldwide [14]. SNPs from RNA-seq data were identified using the Genome Analysis Toolkit (GATK, v.4.1.9.0) [35] and further imputed using the imputation panel via Beagle 5.1 program [36]. For molQTL mapping, both linear regression models and linear mixed models accounting for genetic relatedness as random effects were employed using tensorQTL (v.1.0.4) [37] and rMVP (v.1.0.8) [38], respectively. The top 5 genotype principal components (PCs) and 10 probabilistic estimation of expression residuals (PEER) factors were included as covariates to account for population stratification and hidden factor effects. Only *cis-*molQTL, i.e. SNPs located within 1 Mb upstream and downstream of a gene’s transcription start site (TSS), were considered. To fine-map the molQTL, a stepwise regression procedure in tensorQTL (v.1.0.4) was performed.

### The potential regulatory mechanisms underlying molQTL

To explore the potential regulatory mechanisms underlying molQTL, 257 epigenomes were obtained [20], consisting of genome-wide profiles of 4 histone modifications (H3K4me1, H3K4me3, H3K27ac, and H3K27me3), CTCF, assay for transposase-accessible chromatin using sequencing (ATAC-seq), and deoxyribonuclease sequencing (DNaseSeq). The mark signals (bigWig) of each sample were created using bamCompare, and *Z*-scores were normalized with the scipy.stats.zscore function in Scipy. The *Z*-score-normalized mark signals (bigWig) were visualized in IGV [39]. A total of 15 chromatin states, including TssA, TssAHet, TxFlnk, TxFlnkWk, TxFlnkHet, EnhA, EnhAMe, EnhAWk, EnhAHet, EnhPois, ATAC_Is, TssBiv, Repr, ReprWk, and Qui, were predicted by ChromHMM and named according to the combinations of epigenome modifications and enrichments around the TSS of genes [20].

### Single-cell RNA-seq data analyses

The Seurat package (v4.1.1) [40] was used for cell-type identification and subsequent analysis. We filtered out low-quality cells, including those with an exceptionally low number of detected genes per cell (fewer than 200) and those with a high proportion of reads aligned to mitochondrial genes (over 20%). Potential multiplets, characterized by aberrantly high gene counts per cell, were eliminated. The Harmony method [41] was employed to remove batch effects. Cell-type markers were defined as genes expressed in at least 25% of cells and significantly overexpressed in the cluster, with a log_2_ fold change (log_2_FC) > 0.25 and a false discovery rate (FDR)-corrected *P*-value < .01. Each cluster was assigned a cell type identity based on known marker genes.

### Identification of context-dependent molQTL

The context-dependent *cis-*eQTL, including sex-biased eQTL, cell type-biased eQTL, and tissue-biased eQTL, were identified by fitting a linear additive model with interaction term: *y*=*g* + *i* +*i × g*+ *c* + *e*, where *y* indicated gene expression value, *g* indicated the additive effect of genotype, *i* indicated the effect of the considered context, such as sex, cell type compositions, or tissues, *i × g* was the interaction effect, *c* was the effect of covariates, including 5 PCs and 10 PEER factors, and *e* was the residual error. The context-dependent *cis-*eQTL with FDR-corrected *P*-value < .01 were considered statistically significant.

### Associations of molQTL with complex traits

**Figure 1.**
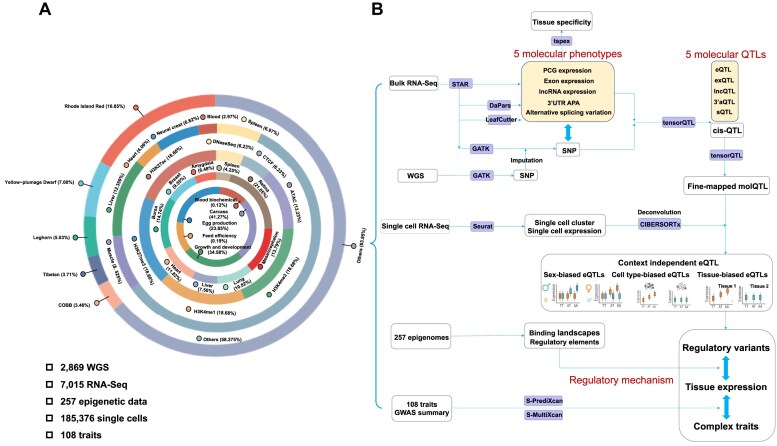
The summary of datasets and pipelines involved in the ChickenGTEx portal. (**A**) The datasets involved. From the outermost to the innermost, the rings represent the breed proportions of 2869 whole-genome data, the tissue proportions of 7015 RNA-seq data, the proportions of different epigenetic markers of 257 epigenetic data, the tissue-type proportions of 185 376 single-cell data, and the proportions of different categories among 108 traits. (**B**) The illustrated pipelines.

To interpret the regulatory effect of molQTL on complex traits, a total of 108 chicken complex traits, ranging from growth, development, and egg production to feed intake and efficiency, were interrogated. Leveraging the GWAS summary statistics of 108 traits, single- and multi-tissue transcriptome-wide association studies (TWAS) were performed using S-PrediXcan and S-MultiXcan in MetaXcan, respectively. The gene-trait associations with FDR-corrected *P*-value < .05 were considered significant. The datasets and pipelines involved in this study are summarized in Fig. 1.

### Database contents and features

The ChickenGTEx portal harnesses a V2G2T framework that integrates pan-tissue molQTL [14], regulatory elements [20], single-cell transcriptomic landscapes [21–27], and context-dependent regulatory variations [14], empowering users to efficiently access, explore, and utilize the integrative genomics resource to decode the molecular and cellular mechanisms underlying complex traits and phenotypic diversity. Key features of the portal encompass: (i) a high-quality 123-breed genotype imputation panel; (ii) five types of molecular phenotypes profiled across 28 tissues; (iii) approximately 2.2 million regulatory variants (*cis-*molQTL) significantly associated with these molecular phenotypes across tissues; (iv) 185 376 single-cell gene expression profiles across 191 cell clusters spanning 9 tissues; (v) 1956 context-dependent molQTL; (vi) 289 epigenetic profiles and 15 chromatin states across 23 tissues; and (vii) potential causative variants and regulatory mechanisms associated with 108 economically important traits. The ChickenGTEx portal will serve as a valuable resource to advance our understanding of fundamental biology, vertebrate evolution, and trait architecture. It will also facilitate precision breeding in poultry and inform translational research in comparative genomics and human biomedicine.

### Molecular phenotypes

**Figure 2.**
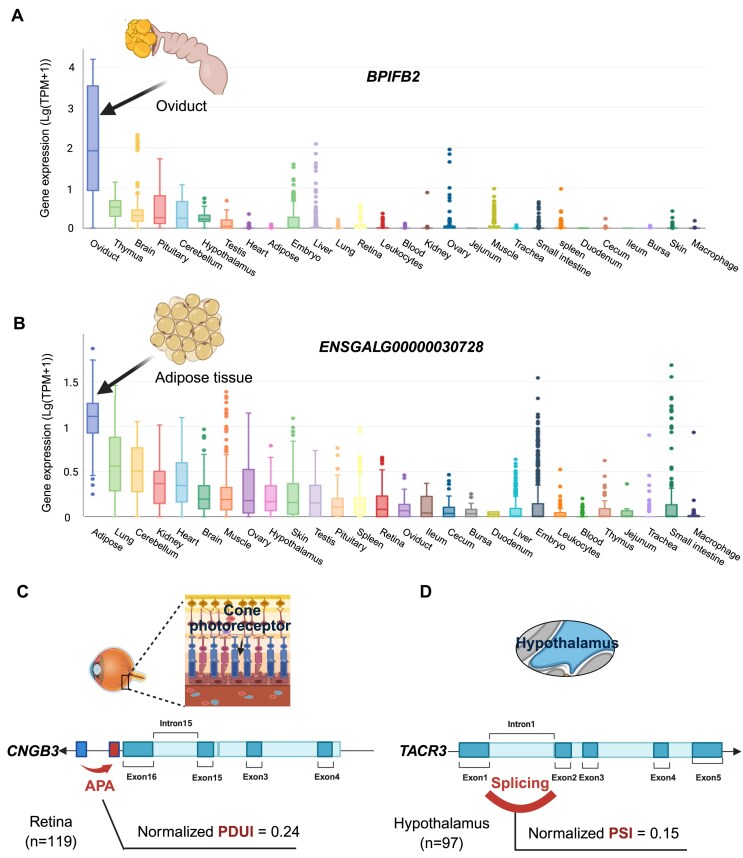
The illustrated molecular phenotypes across 28 tissues. (**A**) PCG expression. (**B**) lncRNA expression. (**C**) 3′UTR APA. (**D**) ASV.

The ChickenGTEx portal provides profiles of five types of transcriptomic phenotypes derived from 7015 bulk RNA-seq datasets, including the expression of PCGs, exons, and lncRNAs, as well as patterns of APAs and ASVs, across 28 tissues. Gene expression patterns across tissues were characterized, and tissue specificity was evaluated using eight scoring metrics that reflect both shared physiological functions and unique biological characteristics of the tissues. For example, *BPIFB2* and *BPIFB3*, encoding lipid transfer/lipopolysaccharide-binding proteins, were highly expressed in the oviduct (Tau = 0.94), whereas an lncRNA (ENSGALG00000030728) with unknown function exhibited relatively high expression levels in adipose tissue (Tau = 0.94) (Fig. 2A and B).

APA is a widespread post-transcriptional regulatory mechanism that modulates messenger RNA stability and translational efficiency, contributing substantially to the molecular basis of human complex traits and diseases [2]. The ChickenGTEx portal accommodates 15 847 identified APA events across 28 tissues and provides the average normalized PDUI values at the polyA position for each tissue. Among these, 3819 APA events are shared across all examined tissues, indicating their conserved regulatory role. For example, *BRD8* exhibited the most significant average normalized PDUI values across tissues. This gene encodes a protein that interacts with the thyroid hormone receptor (THR) in a ligand-dependent manner and enhances thyroid hormone-dependent activation. Given that THR plays critical roles in vertebrate metabolism and reproductive timing, the APA event in *BRD8* may effectively regulate reproduction and growth in chickens. Notably, its human ortholog has been significantly associated with body mass index (BMI) and age at menarche [42]. Furthermore, the portal contains 993 tissue-specific APA events, ranging from 0 in the small intestine (9 RNA-seq datasets) to 200 in the retina (120 RNA-seq datasets). The retina is particularly noteworthy in avian biology (especially in chickens) due to its distinct APA landscape. For instance, a retina-specific APA event was identified in the *CNGB3* gene (Fig. 2C). Activation of this gene can lead to the opening of the cation channel, causing depolarization of rod photoreceptors, which is essential for generating light-evoked electrical responses in cones [43]. The human *CNGB3* gene has been strongly associated with body height, BMI, and reproductive timing [42].

ASV represents another critical mechanism for eukaryotic transcriptional expression and cellular function. The ChickenGTEx portal provides significant ASV events, listing the average normalized PSI values for each gene intron and its intron clustering. A collection of 677 tissue-specific ASV events are highlighted. For instance, a frequently occurring intron 1 retention is detected on the *TACR3* gene specifically in the hypothalamus (Fig. 2D). *TACR3* encodes the receptor of neurokinin B, which regulates pulsatile gonadotropin-releasing hormone-induced gonadotropin secretion, responsible for pubertal activity and progression [13]. The ASV of *TACR3* may shed light on the molecular mechanisms underlying the alternative age of puberty in chickens.

### Regulatory variants associated with molecular phenotypes (*cis*-molQTL)

**Figure 3.**
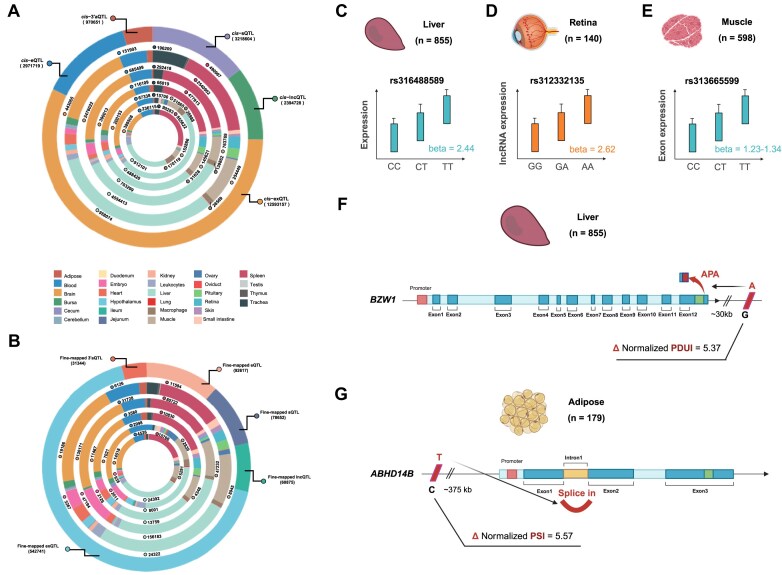
Profiles of molQTL and fine-mapped molQTL. (**A**) The summary of molQTL. The outermost ring indicates the relative prevalence of the five distinct types of molQTL. The inward rings indicate the tissue distributions for eQTL, exQTL, lncQTL, 3′aQTL, and sQTL in turn. (**B**) The summary of fine-mapped molQTL. (**C**) An example of eQTL. (**D**) An example of lncQTL. (**E**) An example of exQTL. (**F**) An example of 3′aQTL. (**G**) An example of sQTL.

The ChickenGTEx portal catalogues 2 971 719 *cis*-eQTL associated with PCG expression across 28 tissues, alongside 2 394 728 *cis*-lncQTL for lncRNA expression, 12 593 157 *cis*-exQTL for exon expression, 970 651 *cis*-3′aQTL for 3′UTR APA, and 3 218 604 *cis*-sQTL for ASV (Fig. 3A). Since these associations may be influenced by linkage disequilibrium between SNPs, the ChickenGTEx portal incorporates a variety of potential causal variants influencing molecular phenotypes, including 92 617 fine-mapped eQTL, 60 875 fine-mapped lncQTL, 542 741 fine-mapped exQTL, 31 344 fine-mapped 3′aQTL, and 78 652 fine-mapped sQTL (Fig. 3B). These datasets enable users to better understand how genomic variants regulate gene expression and post-transcriptional modification. For instance, variant rs316488589 was highly associated with *LCOR* expression in the liver, with the T allele increasing expression by 2.44 units of TPM (Fig. 3C). *LCOR* is a gene implicated in body height regulation. Similarly, rs312332135 modulated lncRNA *ENSGALG00000051591* expression in the retina (Fig. 3D), and rs313665599 affected *DMD* exon usage in the muscle (Fig. 3E). Interestingly, a rare variant, rs731959666, located ∼30 kb downstream of *BZW1*, a gene encoding a translation initiation regulator, led to an APA event with the A allele increasing normalized PDUI value by 5.37 in the liver (Fig. 3F). Additionally, a common variant 12_3166845, located ∼375 kb upstream of *ABHD14B*, showed a significant association with intron 1 retention, with the T allele increasing the normalized PSI value by 5.57 in adipose tissue (Fig. 3G).

### Regulatory mechanisms of genomic variants on molecular phenotypes

**Figure 4.**
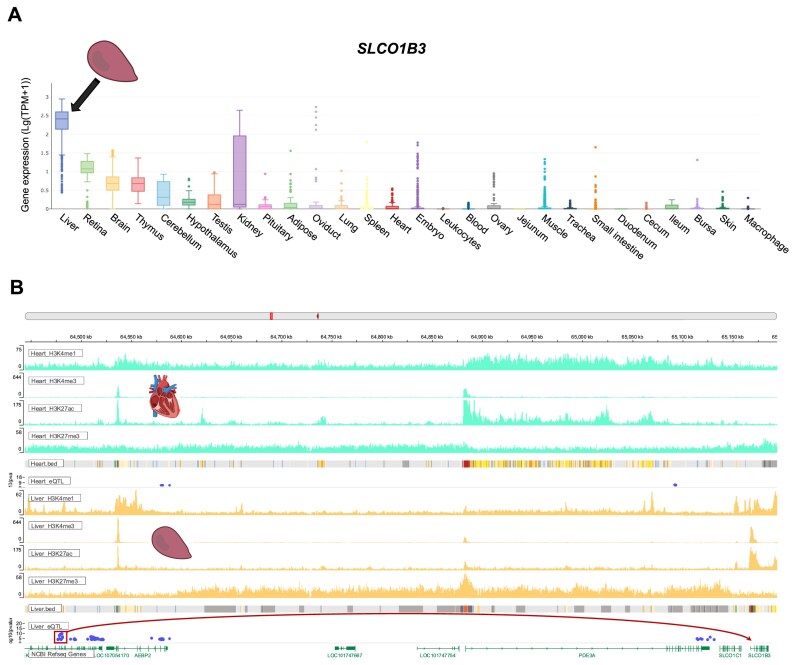
The regulatory mechanisms of a genomic variant on the liver-specific expression of *SLCO1B3* gene. SNP rs738808334 resides within a liver-specific H3K4me1 binding peak and a predicted enhancer, exhibiting a significant association with the liver-specific expression of *SLCO1B3*. Consistently, the gene body of *SLCO1B3* is enriched for binding peaks of active chromatin marks in the liver.

To facilitate the exploration of regulatory mechanisms by which molQTL influences transcriptional diversity, the ChickenGTEx portal provides access to 257 epigenomic profiles across 23 tissues, including ChIP-seq data for five active chromatin marks (i.e. H3K4me3, H3K4me1, H3K27ac, ATAC, and DNase), the repressive polycomb mark H3K27me3, and the architectural protein CTCF, as well as 15 chromatin states derived from these epigenomic signatures [20]. By integrating tissue-dependent chromatin states, epigenetic binding landscapes, and regulatory associations between genomic variants and molecular phenotypes, the portal enables inference of potential regulatory mechanisms of molQTL. Taking an example, SNP rs738808334 resided within a liver-specific H3K4me1 binding peak and a predicted enhancer, exhibiting a significant association with the liver-specific expression of *SLCO1B3*. Consistently, the gene body of *SLCO1B3* was enriched for binding peaks of active chromatin marks in the liver (Fig. 4). Collectively, the ChickenGTEx portal facilitates the interpretation of the regulatory mechanisms of genomic variants on molecular phenotypes.

### Single-cell expression profiles

**Figure 5.**
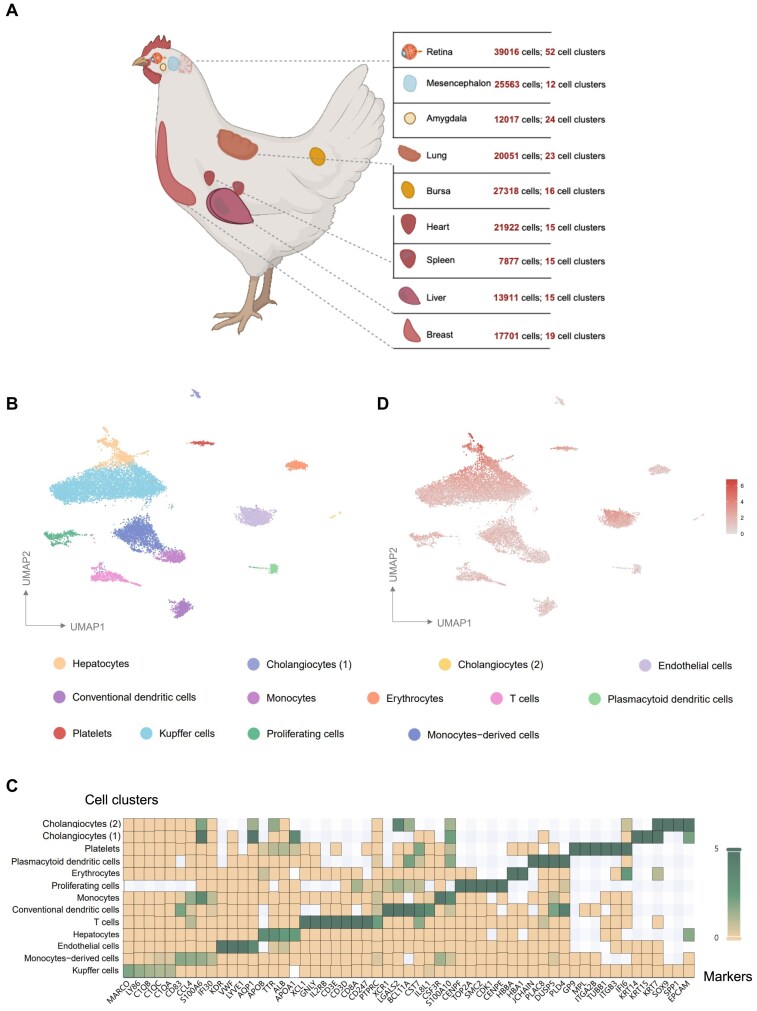
Single-cell expression profiles across nine distinct chicken tissues (*n* = 185 376). (**A**) The dataset includes single-cell expression profiles from nine tissues: spleen, liver, heart, breast muscle, retina, bursa of Fabricius, lung, amygdala, and mesencephalon, with cell counts of 7877, 13 911, 21 922, 17 701, 39 016, 27 318, 20 051, 12 017, and 25 563, respectively. (**B**) UMAP (Uniform Manifold Approximation and Projection) plot depicting cell clusters in the liver as a representative tissue example. (**C**) Gene expression profiles of cell markers across cell types. (**D**) Gene expression profiles of *ALB* in hepatocytes.

Building on lessons from the human GTEx project, cell type composition has emerged as a critical factor in understanding gene regulatory mechanisms within tissues [14]. Identifying causal variants associated with molecular phenotypes and complex traits requires deciphering regulatory relationships at the resolution of individual cell types. The ChickenGTEx portal hosts 185 376 single-cell expression profiles, encompassing 45 402 features and 191 cell clusters across nine distinct tissues—liver, breast muscle, bursa, heart, spleen, lung, retina, amygdala, and mesencephalon. These range from 12 cell clusters and 25 563 single-cell expression profiles in the mesencephalon to 52 cell clusters and 39 016 single-cell expression profiles in the retina (Fig. 5). The portal offers detailed information on cell type composition across these tissues and provides gene expression hierarchy at the cell-type level. For instance, the canonical liver-specific gene *ALB* shows exclusively high expression in hepatocytes, exemplifying cell-type-specific transcriptional regulation (Fig. 5).

### Context-dependent molQTL

The regulatory effects of genetic variants on molecular phenotypes and complex traits can vary across different biological contexts, such as sex, cell type, and tissue. To elucidate context-dependent regulatory mechanisms, the ChickenGTEx portal enumerates 1194 sex-biased eQTL, 703 cell type-biased eQTL, and 59 tissue-biased eQTL.

**Figure 6.**
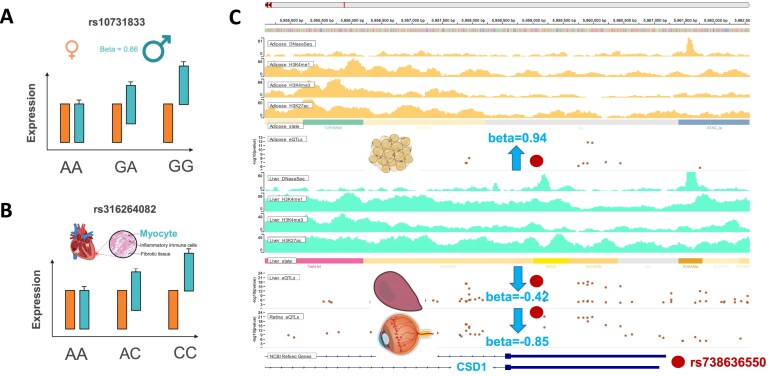
Illustration of context-dependent molQTL. (**A**) A sex-biased eQTL regulating *CPS1* expression in liver. (**B**) A cell-type-biased eQTL regulating *PPP1R3D* expression in heart. (**C**) A tissue-biased eQTL regulating *CSD1* expression in adipose.

Sex-biased eQTL highlight distinct regulatory effects of genetic variants between males and females. For instance, the portal identified a sex-biased *cis*-eQTL (rs10731833) that regulates *CPS1* expression in the liver, with the G allele associated with increased *CPS1* expression in males (*P* = 4.72 × 10^−6^), but not in females (Fig. 6A). *CPS1* is a critical mitochondrial gene involved in protein and nitrogen metabolism and participates in the urea cycle—a pathway known to exhibit sex-specific variation in nitrogen metabolism. Cell type-biased eQTL reveal the cell type specificity of genetic regulatory effects. For example, the C allele of rs316264082 regulates *PPP1R3D* expression in a manner dependent on cardiomyocyte abundance in heart tissue (Fig. 6B). Tissue-biased eQTL facilitate the interpretation of tissue-specific and even opposing regulatory effects of genomic variants. For instance, the T allele of variant rs738636550 yields increased expression of *CSD1* by 0.94 units in adipose tissue but decreased expression by 0.42 and 0.85 units in the liver and retina, respectively (Fig. 6C). *CSD1* encodes a cytosolic copper/zinc superoxide dismutase that detoxifies superoxide radicals, suggesting that this variant may contribute to distinct oxidative stress responses across tissues.

### Regulatory effects of variants on tissue-specific gene expression and complex traits

**Figure 7.**
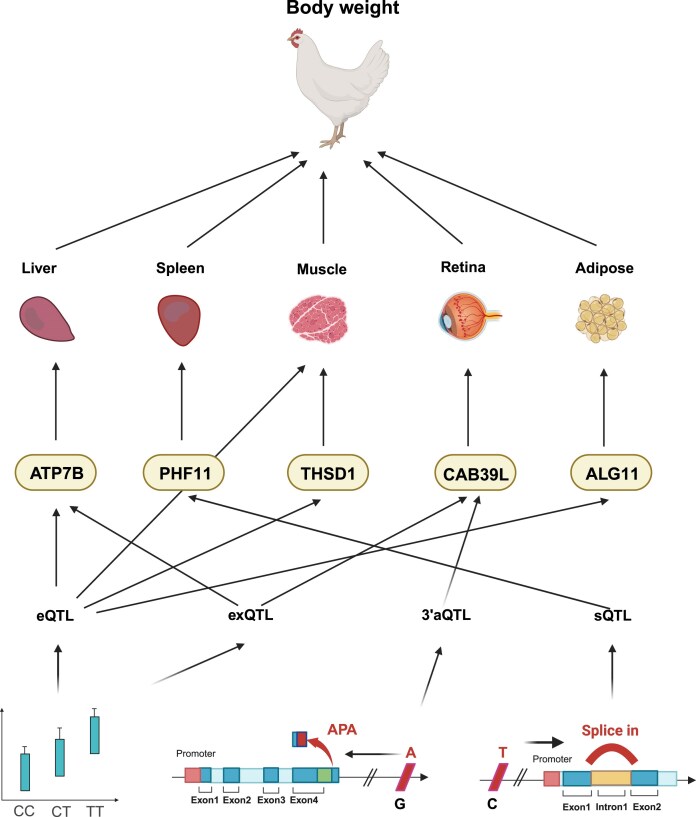
The regulatory hierarchies of complex traits at the levels of variants, genes, and tissues were revealed by TWAS.

To elucidate how regulatory variants affect gene expression across tissues and contribute to complex traits in chickens, the ChickenGTEx portal provides 96 386 significant gene-trait associations. These complex traits include those related to growth, development, egg production, feed intake, and efficiency. Based on these associations, the portal enables users to infer the regulatory hierarchies of genetic variants underlying 108 complex traits—that is, to explore their genetic architectures. Taking an example, a cluster of body weight traits—including body weight at 6 weeks, 8 weeks, and so forth—was orchestrated by eQTL and exQTL of *ATP7B* in liver, the sQTL of *PHF11* in spleen and its eQTL in muscle, the eQTL of *THSD1* in muscle, the exQTL and 3′aQTL of *CAB39L* in retina, and the eQTL of *ALG11* in adipose tissue. Together, these regulatory variants form intricate networks shaping the genetic basis of body weight traits (Fig. 7).

### Discussion and future developments

The ChickenGTEx portal leverages a robust V2G2T framework by integrating pan-tissue molQTL [14], regulatory elements [20], single-cell transcriptomic landscapes [21–27], and context-dependent regulatory variations [14]. This integrative architecture enables researchers to efficiently access, explore, and utilize the resource to elucidate the molecular and cellular mechanisms underlying complex traits and phenotypic diversity in chickens. By offering this wealth of information, the portal provides critical insights into the functional significance of non-coding variants, particularly their roles in modulating molecular phenotypes and complex traits across tissues and cell types. By bridging genotypes with regulatory function and phenotypes, the ChickenGTEx portal serves as a foundational resource for advancing mechanistic understanding in a variety of research fields ranging from molecular biology and vertebrate evolution to applied breeding and translational research. Future directions include the continual integration of high-resolution multi-omics datasets—especially single-cell profiles and data from well-designed experiments—as well as improvements to web-based analytical tools, all tailored to support diverse user needs through intuitive interfaces and rigorously curated data. We envision the ChickenGTEx portal as a pivotal platform that will accelerate discovery across both fundamental and applied life sciences.

## Data Availability

The ChickenGTEx portal can be freely accessed at http://chicken.farmgtex.org.
